# Review of "*Cryptosporidium *and cryptosporidiosis" by Ronald Fayer and Lihua Xiao (eds.)

**DOI:** 10.1186/1756-3305-1-47

**Published:** 2008-12-30

**Authors:** RC Andrew Thompson

**Affiliations:** 1 School of Veterinary and Biomedical Sciences, Murdoch University, Perth, Western Australia, Australia

## Review

With the tremendous upsurge in research activity on *Cryptosporidium *in the mid 1990's, the only comprehensive reference work available at the time was the first edition of this book. As such, it became the 'bible' for many researchers starting out on investigations on this remarkable parasite, and stood the test of time for many years. Today, the volume of research articles on *Cryptosporidium *is enormous and not surprisingly, for an edited text to provide a comprehensive and up to date resource that is representative of current developments is a tall order! The chapters on genomics and biochemistry are valuable additions, and the host assemblage chapters provide a useful resource. However, I do not feel this second edition will find its place on as many of the bookshelves of *Cryptosporidium *researchers as did the first edition. Why? The field is moving so quickly that this new edition cannot be said to reflect the current status in some fields of *Cryptosporidium *research. The most recent literature cited is two years old, although, I know from personal experience with edited volumes how difficult it is to ensure that the final publication is up-to-date in such a fast moving field. However, recent developments in surface receptors, the host parasite interface, developmental biology, phylogenetic affinities and how these relate to *Cryptosporidium*'s unique biology, are given only cursory mention but are likely to dominate thinking over the next few years, which promise to be an exciting period of *Cryptosporidium *research.

There are 20 chapters of varying length that cover the biology of the organism, genomics and biochemistry, epidemiology and transmission (via food and water), diagnosis, immune responses and clinical disease, *Cryptosporidium *in specific host assemblages, and *in vivo *and *in vitro *maintenance. There is no contents page listing specific chapters and their contributors which I find most unusual in such a book, and thus it is difficult to find your way around. This is exacerbated by the type of paper used and quality of some of the figures that have been re-printed from the first edition.

This is a useful purchase for libraries but individual researchers, who have a copy of the previous edition, can be better informed by the frequent and varied reviews that have, and continue to, appear on a regular basis.

## Competing interests

Currently co-editing a conference proceedings on *Giardia *and *Cryptosporidium*.

